# Supplementary material for: Prevalence of malaria and associated factors among under-five children in Sherkole refugee camp, Benishangul-Gumuz region, Ethiopia. A cross-sectional study
Source: PLoS One. 2021 Feb 19;16(2):e0246895. doi: 10.1371/journal.pone.0246895 (PMC7894890; doi:10.1371/journal.pone.0246895)
Supplement: Attachment — Submitted filename: Response for reviews pols mala.docx [file pone.0246895.s002.docx]

Response for reviews

First of all, I would like to thank you for all reviewer for their constructive comments. and I incorporate all comments in the manuscript. The comment give for me is very constrictive and important. so, I try to address all the issue that are given to me, but still now if there is anything that must be amended, I am ready to receive your feedback thank you. But for some issue that needs more justification I wrote here under with.

The authors collected data by interviewing the mothers or caregivers and biological specimen from under five children. What is the outcome or dependent variable and which variables are independent variables?

The outcome variable is malaria (positive/negative) whereas, Independent variables were socio-demographic characteristics, housing condition /refuge/, ITN condition and availability, ITN utilization, IRS, presence of stagnant water, outdoor stay at night, and Health information about malaria were included in the questionnaires

What are the contents of the questionnaire? How was it collected? Is it face to face, self-administered? paper-based or electronically?
The authors collected 5 microliters of whole blood, from where and how? It will do the audience better if the author can explain the step by step process, what type of bottle was used to collect the blood? How did author or laboratory scientist process the blood for testing? What kind of RDT kits was used? What was the sensitivity or specificity of the test by the manufacturer? How long was the blood kept before testing or was it tested immediately? How did test differentiate the species of Plasmodium?

A finger prick was performed using sterile lancets, and using capillary tube, 5 micro liters of whole blood was drawn from each child. The blood sample was tested immediately using Care Start^TM^ Malaria RDT which detect histidine-rich protein 2 (HRP2) of Plasmodium falciparum and plasmodium lactate dehydrogenase (pLDH) of Plasmodium vivax. According to the manufacturer, the sensitivity and specificity of the RDT was 98% and 97.5% respectively.

77 (49.7%) were with greater than or equal to two under five children.” What do this mean to the authors?nThis mean that when the households had two and above />=2/ under-five children

To report malaria prevalence, the authors need to provide denominators for clarity in this statement, “The proportion of plasmodium species was 12 (85.7%) and 2 (14.3%) for Plasmodium Falciparum and Plasmodium Vivax respectively.

The overall prevalence of malaria among under-five children in sherkole refugee camp was 14(**3.9%) (**14/356) (95% CI=2.0-6.2). The proportion of plasmodium species was 12/14 (85.7%) and 2/14 (14.3%) for Plasmodium Falciparum and Plasmodium Vivax respectively. In terms of gender, the prevalence was 6/172 (3.5%) and 8/184 (4.3%) for male and female respectively.

Thank you

Kebadnew
