# Supplementary material for: Prevalence of malaria and associated factors among under-five children in Sherkole refugee camp, Benishangul-Gumuz region, Ethiopia. A cross-sectional study
Source: PLoS One. 2021 Feb 19;16(2):e0246895. doi: 10.1371/journal.pone.0246895 (PMC7894890; doi:10.1371/journal.pone.0246895)
Supplement: Attachment — Submitted filename: Clarification for issues raised on submitted manuscript.docx [file pone.0246895.s003.docx]

**Clarification for issues raised on submitted manuscript**

First of all, I would like to thank you for all reviewer for their constructive comments. and I incorporate all comments in the manuscript, and for some feathers that needs some sort of explanation, I try to reason out here below.

1. The issue of RDT

The type of RDT used to test the blood sample in this study is Care Start^TM^ Malaria RDT.

It detect histidine-rich protein 2 (HRP2) of Plasmodium falciparum and plasmodium lactate dehydrogenase (pLDH) of Plasmodium vivax. So, it can separately detect antigens of P. falciparum and P. vivax.

According to the manufacturer, the sensitivity and specificity of the RDT was 98% and 97.5% respectively.

2. Regarding Health information about malaria

This means caregivers/mothers who have previous health information/awareness about malaria’s mode of transmission and ways of prevention.

3. Insecticide treated net (ITN) utilization was measured by asking the caregivers whether the under-five children slept in ITN during the night preceding the survey or not. ITN condition was assessed by observing the currently used ITN. Caregivers were also asked whether their houses got Indoor Residual Spray (IRS) service in the last six months or not
